# A prediction model for childhood obesity risk using the machine learning method: a panel study on Korean children

**DOI:** 10.1038/s41598-023-37171-4

**Published:** 2023-06-21

**Authors:** Heemoon Lim, Hyejung Lee, Joungyoun Kim

**Affiliations:** 1 College of Nursing, Yonsei University, Seoul, South Korea; 2 College of Nursing, Yonsei University, Mo-Im Kim Nursing Research Institute, Seoul, South Korea; 3 Department of Artificial Intelligence, University of Seoul, Seoul, South Korea

**Keywords:** Endocrinology, Health care, Risk factors

## Abstract

Young children are increasingly exposed to an obesogenic environment through increased intake of processed food and decreased physical activity. Mothers’ perceptions of obesity and parenting styles influence children’s abilities to maintain a healthy weight. This study developed a prediction model for childhood obesity in 10-year-olds, and identify relevant risk factors using a machine learning method. Data on 1185 children and their mothers were obtained from the Korean National Panel Study. A prediction model for obesity was developed based on ten factors related to children (gender, eating habits, activity, and previous body mass index) and their mothers (education level, self-esteem, and body mass index). These factors were selected based on the least absolute shrinkage and selection operator. The prediction model was validated with an Area Under the Receiver Operator Characteristic Curve of 0.82 and an accuracy of 76%. Other than body mass index for both children and mothers, significant risk factors for childhood obesity were less physical activity among children and higher self-esteem among mothers. This study adds new evidence demonstrating that maternal self-esteem is related to children’s body mass index. Future studies are needed to develop effective strategies for screening young children at risk for obesity, along with their mothers.

## Introduction

Over the last four decades, the worldwide prevalence of childhood obesity has rapidly increased^1^. A recent growth trajectory simulation predicted that, by 2025, half the children in the world would be obese^2^. In 2019, a quarter of children and adolescents under the age of 18 in Korea were reported to be overweight or obese; the rate had increased from 21.9% in 2015, which is an increase of 3.9%. During the same period, the increase in overweight and obese adults was 0.6%^3^. In the United States, obesity at the age of 10 years indicates a propensity toward obesity in the future^4^. Moreover, the negative impact on the physical, emotional, and social functions of being overweight and obese in childhood may contribute to worsening health in adulthood^5,6^. These findings support the need to prevent and manage childhood obesity at or before the age of 10 years to decrease adulthood obesity because managing weight is an even greater challenge for obese adults^7^.

The exact etiology of childhood obesity has not been fully explained; instead, it appears that multiple individual, family, and environmental factors are interrelated^5,8–10^. In recent years, it seems that children are increasingly likely to be exposed to a so-called “obesogenic environment,” which includes increased intake of processed food and decreased physical activity^6^. To promote health in children by combating this obesogenic environment, a combined early intervention of nutrition, physical activity, and lifestyle modification is needed^5,11,12^.

Machine learning techniques offer excellent modeling tools that deal with vast amounts of highly complicated data. Furthermore, these techniques make it possible to analyze undefined health problems, thus leading to better decision-making^13^. Obesity in children—from preschoolers to adolescents—has been predicted using machine learning approaches^14–18^. High-quality data on gender, age, weight, disease status from infant health check-ups^8^, pediatric hospitals^14^, and public health center EHR systems^17^ have provided an accurate prediction model with high predictive performance. However, which set of features and algorithms provides the best prediction for childhood obesity is still under investigation. In addition, previously reported prediction models were limited in their ability to explain the relationships between the risk factors and children’s obesity development^15,17,19^. Regarding an obesogenic environment, features related to cultural characteristics, such as parenting style and table manners, need to be considered to obtain the best-performing algorithm for children in different countries^15,17^.

Preschoolers between 3 and 5 years are in a unique period of childhood during which they develop many social behaviors required to actively interact with the environment^20^. At 5 years of age, children begin to learn and establish healthy lifestyles that may continue into their adulthoods^21^; therefore, mothers play an important role in creating the foundation of their children’s lifestyles. Parents’ psychological state has been clearly connected to how they choose to raise their children^22^. A systematic review of parenting style found that parents who were not interested in or involved in the care of their children may cause adverse mental health in children. In one study conducted in Korea, a major factor in determining the parenting style was the mother’s mental well-being^23^. Thus, this study attempted to consider factors related to the mothers, such as depression, self-esteem, and parenting style, to better predict obesity in 10-year-old children.

The availability of machine learning algorithms may meaningfully improve screening of children who are at a greater risk of becoming obese by simultaneously considering the child’s lifestyle and maternal psychological status. This study’s specific aims are: (1) to develop a prediction model to estimate obesity in 10-year-old children using the national panel data and (2) to identify the risk factors affecting obesity in those children. The findings of this study would help develop a more effective prevention strategy focused on children at high risk for obesity.

## Methods

### Design and data

This secondary data analysis study used the Panel Study on Korean Children (PSKC) conducted by the Korea Institute of Child Care and Education. All data from this study are publicly available. The PSKC is a longitudinal survey that collected data regarding children’s demographic characteristics, family background, and factors related to a child’s growth and development, including parenting style and maternal psychological status. Children born between April and July 2008 were recruited from 30 hospitals that were sampled using a stratified multistage sampling technique. The first survey involved a face-to-face interview and was conducted in the hospital when the child was born. Then, subsequent surveys were conducted annually using self-administered questionnaires and face-to-face interviews. A total of 2078 families participated in the first survey. By the 11th survey, 1434 families continued to participate annually; thus, the sample attrition was minimal.

For this study, we selected maternal and childbirth data obtained from the 1st survey, lifestyle and eating habits of children at 5 years of age from the 6th survey, and the obesity status of children at 10 years of age from the 11th survey. The first criterion for including a child in our study was that BMI measurements at both 5 and 10 years of age were available. One hundred and fifty-four families provided no data on the 5- or 10-year-olds’ BMIs, and 95 families provided none of the data needed for this study (e.g., eating habits, maternal depression, and parenting style). Data from 1185 children who met inclusion criteria were used in the final analysis. Then, they were randomly split into either the training data group (70%) for the prediction model. Validation of the predictive model developed by the training data set used a method of preserving 30% of the entire data as test data by random assignment before developing the model^18^. The sample size in this study satisfied the minimum number of sample that calculated for the logistic regression analysis^24^.

### Features for developing a prediction model

We utilized data regarding the following factors: demographic information (gender, birth weight); lifestyle (sleep time, eating habits); physical activity (sedentary time, time spent in indoor play, and outdoors); obesity status (5-year-old and 10-year-old BMI); and maternal data (education level, BMI, smoking, depression, self-esteem, and parenting style). Table 1 presents the variables considered to be risk factors that also were used to develop a proper prediction model. Studies that investigated predictive models using longitudinal cohort data found that birth weight, BMI, eating habits, and educational level of mother were significant predictors. An reviews reported BMI of children and self-esteem of the mother to be significant factors for children’s obesity^22^. For this study, we included those variables to develop a prediction model. We conducted a regularization process based on the average and standard deviation of the variables for feature selection and prediction model development using LASSO.

**Table 1:** Variables used for developing the prediction model for childhood obesity.

Category	Variable
Child	
Demographics	Gender^17^, Birth weight^8,9^, BMI at 5 years old^17,21^
Lifestyle	Sleep hour^9^, Eating habits^8,9^ (regularity, eating speed, frequency of snack consumption), Activities^33^ (sedentary time, time spent playing indoors and outdoors)
Mother	
Demographics	Education level^8^, BMI^9^, Smoking^18^
Psychological factors	Depression^38^, Self-esteem^22^
Parenting style	Warm parenting style^32^, Control parenting style^32^
Social economic status	Income^9^

*BMI* Body Mass Index.

The least absolute shrinkage and selection operator (LASSO) is one method based on model reduction. The main concept of LASSO is to construct a penalty function that shrinks the regression coefficient of each variable to a certain range. Variables representing 10-year-old BMI are obtained by removing variables with a coefficient of 0 and considering variables with high correlations among predictors. Therefore, coefficients are optimized and relatively unimportant variables are excluded.

### Outcome

The primary outcome for this study was obesity at 10 years of age and was classified as a binary outcome. Overweight and obesity in children was defined as having a BMI ≥ 85th percentile for age and sex based on the 2017 Korean National Growth Chart published by the Korea Centers for Disease Control and Prevention (KCDC)^25^.

### Model development

The classification of data decided class membership ${y}^{new}$ of unknown data (${x}^{new}$) based on training data set $D=\left({x}_{1},{y}_{1}\right),\ldots , ({x}_{n},{y}_{n})$, where ${x}_{i}=({x}_{i1}, {x}_{i2}, \ldots , {x}_{ip})$ is a p-dimensional feature and ${y}_{i}$ is the membership of the i-th subject. We considered classification as being a class label (y) of either 0 or 1^26^. The classification algorithm using logistic regression is the predictive analysis algorithm that assigns observations to an individual set of classes. We used class probabilities to predict the binary outcome based on 10-year-old BMI.

We classified children as being obese if they had a predicted value greater than the childhood obesity threshold based on logistical regression estimates^27^.

$$log\; log \left[\frac{P\left({x}_{i}\right)}{1-P\left({x}_{i}\right)}\right] ={\beta }_{0}{+\beta }_{1}{x}_{i1}+{\beta }_{2}{x}_{i2}+\cdots +{\beta }_{p}{x}_{ip}.$$

The threshold value was determined to maximize the Youden index (J = sensitivity + specificity − 1) and to represent the optimal predictive performance among the predictive performance, which changes according to the threshold value of the classification model that is generated based on the test data, as shown below^28^.

$${\widetilde{J}}_{E}={max}_{c}\left\{\widehat{F}\left(c\right)=\widehat{G}\left(c\right)\right\}, c\in [{x}_{m-j+1},\ldots ,{x}_{m}, {y}_{n-k+1},\dots ,{y}_{n].}$$

### Ethical approval

This study was exempted from approval by Severance Hospital Ethics Committee, Seoul, South Korea (No. 2022-2254-001). Anonymized data were obtained from the PSKC, and this study thoroughly followed the manual recommended by the PSKC^29^.

### Statistical analyses

All statistical analyzes were performed in R (version 4.1.3) program. To identify the factors that could accurately predict childhood obesity, the LASSO framework was used with glmnet R packages. Because the LASSO analysis merits control of the sparsity of the independent variables by adding a regularization parameter, this method was selected to test the association between the selected factors and 10-year-old BMI. The binary classification threshold followed the Youden index. The receiver operating characteristic curve (ROC) and the area under the ROC curve (AUC) were also calculated to validate the performance of the predicting model with pROC and ROCR R packages.

## Results

### Characteristics of the children and mothers with the selected features

After pre-processing, a total of 1185 children were included. Of those, 370 were selected for the internal validation process. Out of 18 features, 10 were selected for the final prediction model after a feature selection process. The characteristics of children and mothers in the training and testing sessions are presented in Table 2, along with the selected features used for developing the model. Based on the training data set, the percentages of boys and girls were 51.5% and 48.5%, respectively. The mean 5-year-old BMI was 15.88 (SD 1.72), and the mean sedentary time was 1.37 h (SD 0.85). The mean time spent in indoor play was 2.04 h (SD 1.04), while 1.14 (SD 0.78) hours were spent in outdoor activities per day. Regarding maternal characteristics, the mean maternal BMI was 21.22 (SD 3.02), and self-esteem was 32.1 (SD 5.68). The number of obese/overweight children at 10 years of age was 204 (25.31%) in the training data set.

**Table 2:** Variables used for the final prediction model derivation and validation (N = 1185).

Variable	Characteristics	Categories	For training (N = 806)		For testing (N = 379)	
			N (%)	Mean (SD)	N (%)	Mean (SD)
Child	Gender	Male	415 (51.49)		193 (50.92)	
		Female	391 (48.51)		186 (49.08)	
	Eating regularity	Very irregular	2 (0.25)		0 (0)	
		Almost irregular	32 (3.97)		14 (3.69)	
		Sometimes regular	189 (23.45)		79 (20.84)	
		Almost regular	443 (54.96)		209 (55.15)	
		Very regular	140 (17.37)		77 (20.32)	
	Eating speed (fast)	Very slow	42 (5.21)		21 (5.54)	
		Slow	223 (27.67)		96 (25.33)	
		Medium	416 (51.61)		214 (56.46)	
		Fast	119 (14.77)		41 (10.82)	
		Very fast	6 (0.74)		7 (1.85)	
	Sedentary time (hour/day)			1.37 (0.85)		1.38 (0.80)
	Time spent playing indoors (hour/day)			2.04 (1.04)		2.10 (1.12)
	Time spent playing outdoors (hour/day)			1.14 (0.78)		1.15 (0.85)
	BMI (5-year-old)			15.88 (1.72)		15.80 (1.59)
Mother	Education level	Under high school	4 (0.50)		1 (0.26)	
		High school	219 (27.17)		104 (27.44)	
		College	226 (28.04)		110 (29.03)	
		University	319 (39.58)		140 (36.94)	
		Over master’s degree	38 (4.71)		24 (6.33)	
	Self-esteem			32.1 (5.68)		31.51 (5.95)
	BMI			21.22 (3.02)		21.14 (2.67)
	Obesity status at 10 years old	Within normal limit	602 (74.69)		290 (76.52)	
		Overweight/obesity	204 (25.31)		89 (23.48)	

*BMI* Body Mass Index.

### Feature selection

Ten features were included in the prediction model after the LASSO analysis. The shrinkage parameter was determined to minimize the negative log-likelihood during the LASSO analysis. Gender, eating habits (regularity and speed), physical activity (sedentary time, time spent in outdoor activities, and indoor play hours), and BMI at 5 years of age were all included in the analysis of the children’s data. Additionally, maternal education level, self-esteem, and BMI were included as the maternal factors.

### Prediction model performance

**Figure 1:**
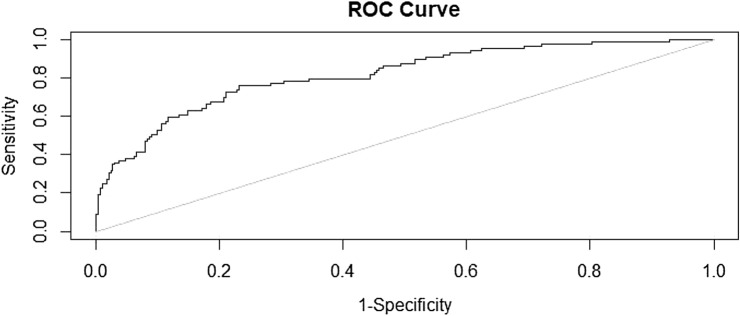
Prediction model’s performance following the ROC curve.

We developed the logistic binary classification model with the threshold (0.26), which was calculated according to the Youden index. The performance of the best prediction model, which utilized the logistic binary classification algorithm with the LASSO, showed 74% accuracy (95% CI [0.69–0.78]), 76% sensitivity, and 73% specificity. Table 3 presents the performance (accuracy, sensitivity, specificity, positive predictive value, and negative predictive value) of the final predictive model for childhood obesity. Figure 1 presents the results of the ROC curve analysis; the AUC result was 0.82.

**Table 3:** Accuracy and predictive capacity of a prediction model for childhood obesity.

Parameter	Probability percentile threshold																		
	5	10	15	20	25	30	35	40	45	50	55	60	65	70	75	80	85	90	95
Accuracy (%)	41.2	53.3	63.1	68.1	74.4	76.8	78.4	81.0	82.6	82.1	82.1	81.5	81.3	80.2	79.4	79.2	78.4	78.1	77.6
95% CI (%)	36.2–46.3	48.1–58.4	58.0–67.9	63.1–72.7	69.7–78.7	72.2–80.9	73.9–82.4	76.7–84.8	78.4–86.3	77.8–85.8	77.8–85.8	77.3–85.3	77.0–85.1	75.8–84.1	75.0–83.4	74.7–83.1	73.9–82.4	73.6–82.2	73.0–81.7
Sensitivity (%)	97.8	93.3	85.4	79.8	77.5	69.7	58.4	49.4	42.7	37.1	37.0	27.0	23.6	18.0	13.5	12.4	9.0	6.7	4.5
Specificity (%)	23.8	41.0	56.2	64.5	73.5	79.0	84.5	90.7	94.8	95.9	96.9	98.3	99.0	99.3	99.7	99.7	99.7	100	100
PPV (%)	28.3	32.7	37.4	40.8	47.3	50.4	53.6	62.0	71.7	73.3	76.9	82.8	87.5	88.9	92.3	91.7	88.9	100	100
NPV (%)	97.2	95.2	92.6	91.2	91.4	89.5	86.9	85.4	84.4	83.2	82.6	81.4	80.8	79.8	79.0	78.7	78.1	77.7	77.3

*CI* confidence interval, *PPV* positive predictive value, *NPV* negative predictive value.

### Predictors for obesity in 10-year-old children

Table 4 presents the binary logistic regression results that tested the relationship between predictors and childhood obesity. Boys, longer sedentary time, less time spent in indoor play, higher 5-year-old BMI, maternal higher self-esteem, and higher maternal BMI significantly increased the likelihood of 10-year-old children developing obesity.

**Table 4:** Best logistic regression model by LASSO.

Variable	Beta	SE	p-value	Odds ratio (95% CI)
Child				
Gender				
Male (reference)				
Female	− 0.45	0.2	0.029	0.64 (0.43–0.95)
Regularity of mealtime				
Very irregular (reference)				
Almost irregular	− 2.37	1.68	0.159	0.09 (0–3.35)
Sometimes regular	− 1.91	1.57	0.225	0.15 (0–4.54)
Almost regular	− 1.84	1.57	0.243	0.16 (0–4.88)
Very regular	− 1.32	1.58	0.405	0.27 (0.01–8.30)
Eating speed				
Very slow (reference)				
Slow	0.25	0.6	0.677	1.28 (0.43–4.62)
Medium	0.63	0.58	0.278	1.87 (0.66–6.57)
Fast	1.05	0.61	0.082	2.87 (0.94–10.51)
Very fast	1.22	1.4	0.385	3.38 (0.20–59.51)
Sedentary time	0.28	0.12	0.021	1.32 (1.04–1.67)
Time spent playing indoors	− 0.28	0.11	0.009	0.76 (0.61–0.93)
Time spent playing outdoors	− 0.03	0.13	0.808	0.97 (0.75–1.25)
BMI at 5 years old	0.73	0.07	< 0.001	2.07 (1.81–2.40)
Mother				
Education level				
Under high school (reference)				
High school	− 0.35	1.22	0.774	0.7 (0.08–15.33)
College	− 0.59	1.23	0.628	0.55 (0.06–12.04)
University	− 0.91	1.23	0.459	0.40 (0.04–8.81)
Over master’s degree	− 0.7	1.3	0.590	0.50 (0.05–11.78)
Self-esteem	0.06	0.02	0.003	1.06 (1.02–1.10)
BMI	0.08	0.03	0.019	1.08 (1.01–1.15)

*LASSO* Least Absolute Shrinkage and Selection Operator, *CI* Confidence Interval, *BMI* Body Mass Index.

## Discussion

To our knowledge, this study is the first to include maternal characteristics in the prediction model for childhood obesity development. Applying machine learning algorithms made it possible to analyze the national panel data, which includes vast information about children’s healthy eating and physical behaviors, maternal psychological status, and parenting style, thereby identifying risk factors for obesity in 10-year-old children. This study’s use of national data revealed that the prevalence of overweight and obesity was 25% among 10-year-olds, which is similar to the prevalence found among school-aged children and adolescents in Korea^3^. Since these obese children will likely become obese adults, the factors contributing to obesity development need to be precisely investigated and managed.

The performance of our final prediction model for childhood obesity was excellent compared to those of previous prediction studies, presenting an AUC ranging from 0.73 to 0.82^17–19^. Our prediction model showed higher performance than the prediction models using children’s food intake and physical activity^26^; vital signs, diagnosis, and laboratory findings^17^; and birth cohort data^18^. These studies also applied the logistic binary classification, as we did. The findings of our study support the idea that mothers’ psychological factors need to be considered to understand childhood obesity development.

It is necessary to compare our machine learning study with similar works with higher prediction findings. One study in Bangladesh developed a prediction model with 97% accuracy using data from 1100 adults^19^. Another prediction model using the ensemble forest method, which predicted 89% of obese adults in India^16^. Both prediction models used cross-sectional data and predicted risk with various features, including weight and height, which are highly correlated when it comes to obesity. Although different types of machine learning methods affect the results predicting obesity^30^, our prediction model using logistic regression has an advantage in terms of determining the likelihood between risk factors and obesity development.

Our prediction models included 10 children-related (gender, eating habits, activity, and BMI at 5 years old) and maternal factors (education level, self-esteem, and BMI). Several machine learning algorithms used to predict obesity reported that gender, previous childhood BMI, and maternal weight and BMI were contributing factors to obesity^15–19^. The gender results in our study mirror those of a study conducted in the United States, which found that more boys than girls were obese^3^. These findings indicate that children’s previous weight, gender, and the BMI status of their mothers should be taken into account in fighting childhood obesity^31^.

Among the 10 factors included in the prediction model, demographics of the children and mothers, children’s activity levels, and maternal self-esteem were significant influencing factors. As expected, intensity and time of physical activity were also significant factors^32^. We selected data concerning different intense activities: outdoor, indoor, and sedentary. In particular, time spent in indoor play that was of moderate intensity, such as playing with a toy and walking around, was a factor contributing to predicting childhood obesity in our model. This was interpreted as indicating that time spent in indoor play has a more varied range for each child compared to time spent in outdoor activities and sedentary time for 5-year-old children. In the prediction model based on the intensity of physical activity, moderate-intensity—not high-intensity—activity was the most significant contributing predictor^33^.

This study is unique in that it includes maternal data in the development of a prediction model. Interestingly, maternal self-esteem, which has not been examined previously, was found to be a contributing predictor of developing childhood obesity. A review of the relationship between self-esteem and parenting reported that higher self-esteem predicted a higher authoritative parenting style, which is characterized by setting clear rules and expectations and solving problems together with a child. This parenting style is high in both warmth and control^22^. Lopez and colleagues found that the authoritative parenting style encourages the intake of healthy food^34^. However, our result demonstrated that higher self-esteem among mothers increased the likelihood of childhood obesity development. As with the results of a study on Korean parenting style, mothers’ self-esteem had a positive interrelated relationship with the warm parenting style that allowed children to choose their own activities^35^. This, in turn, may have influenced dietary behaviors in relation to obesity. In general, maternal self-esteem is known to influence children in many ways. Mothers’ high self-esteem mediates good coping strategies in the face of high stress, and this affects not only eating practices but also children’s healthy behaviors^36,37^.

Several limitations of this study should be noted. Our panel data constituted a relatively small sample size, although these data were longitudinally obtained. Although the data is intended to represent the characteristics of all children and families residing in Korea, there are limitations in considering all of the various factors that affect children's obesity. However, the results are meaningful in that they predicted the factors affecting obesity in 10-year-old children by applying a statistical method. Regression models used to predict obesity and explain association with obesity are one of the basic methods of predictive models and may have limitations in estimating nonlinear relationships. In addition, since the results of this study are specifically drawn from data about children residing in Korea and their families, caution must be exercised when generalizing the results. For future studies, we recommend utilizing a larger sample size and investigating the role of self-esteem in childhood obesity development.

### Conclusions and implications

Based on the national panel data, we developed a prediction model for obesity risk at age 10 that showed good performance. Among those selected from pre-processing, 10 factors (child’s gender, eating habits, activity, 5-year-old BMI, maternal education level, self-esteem, and BMI) were included in the final model. In addition to the previous higher BMI for both children and mothers, children who are boys, who spend less time indoors, and whose mothers have high self-esteem levels are more likely to develop obesity. The findings of this study provide a basis for developing effective strategies to screen children at risk for obesity and develop preventive interventions targeted at those children. Such efforts would help create a less “obesogenic environment,” thus leading to healthier children and healthier future adults.

## Data Availability

The datasets generated and/or analyzed during the current study are available from the corresponding author on reasonable request.
